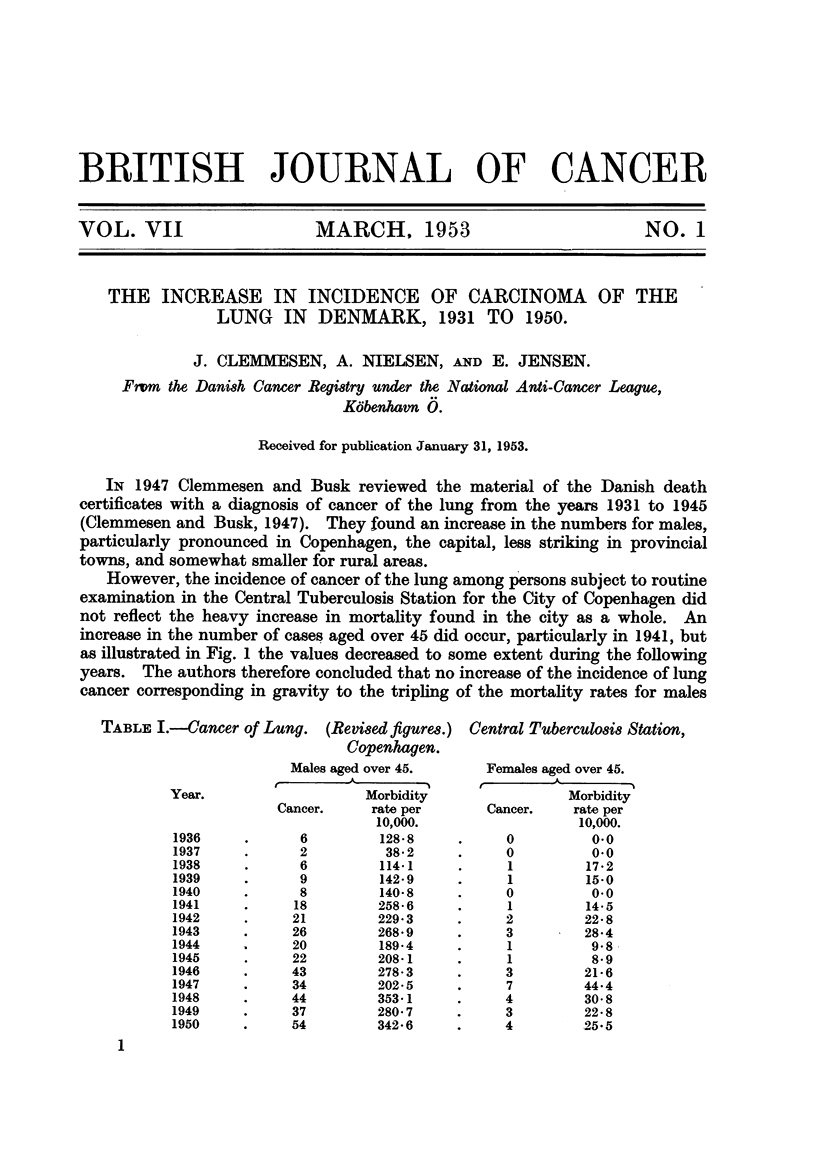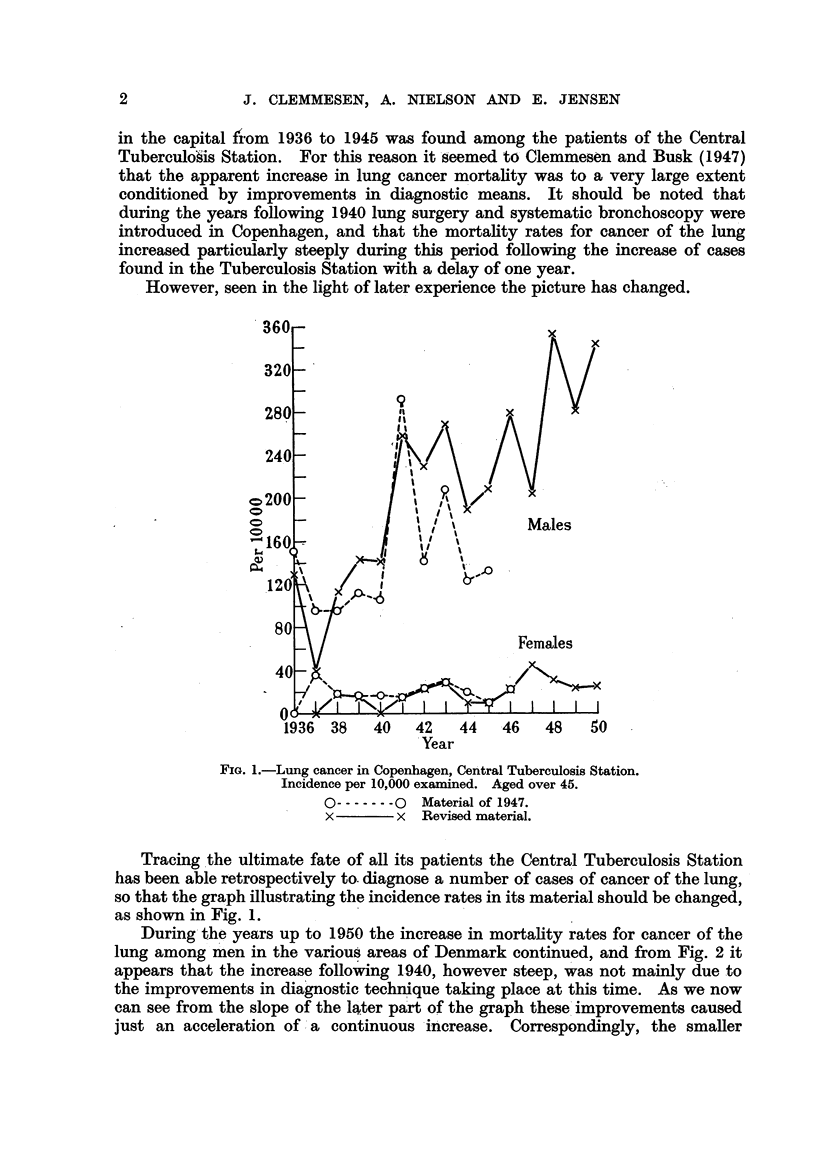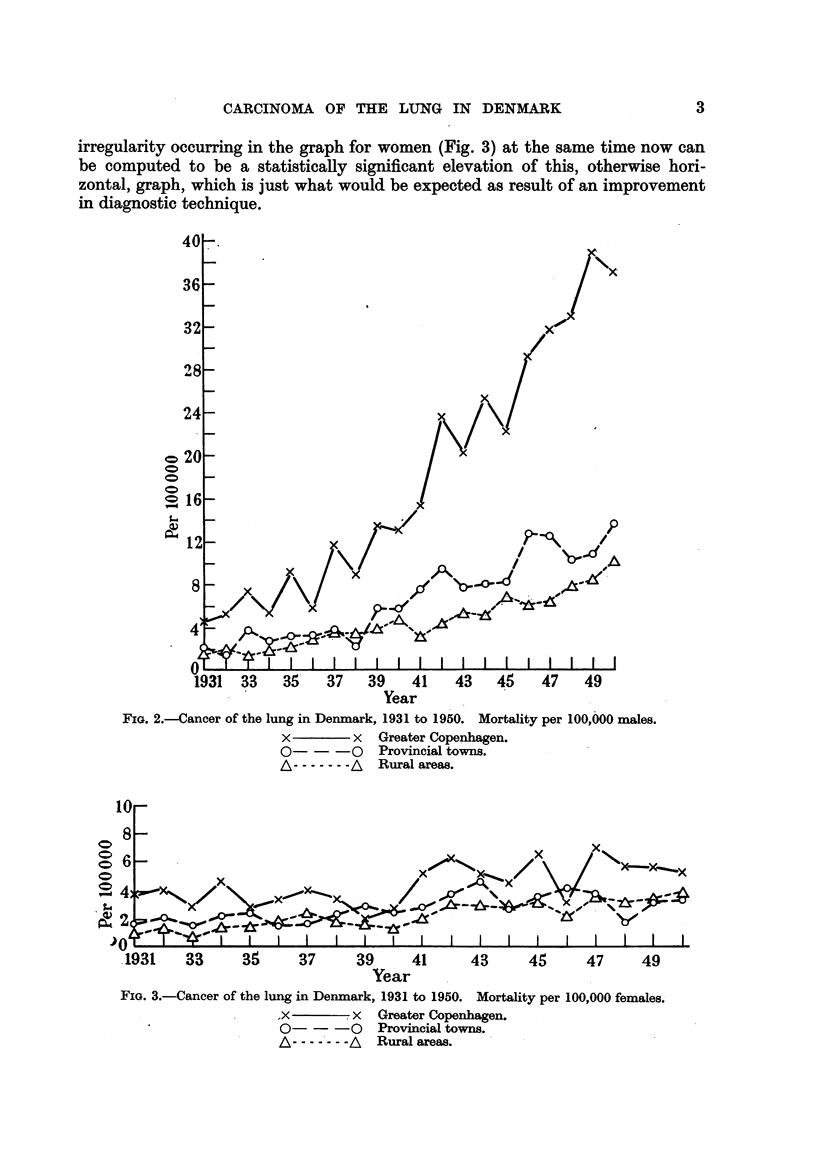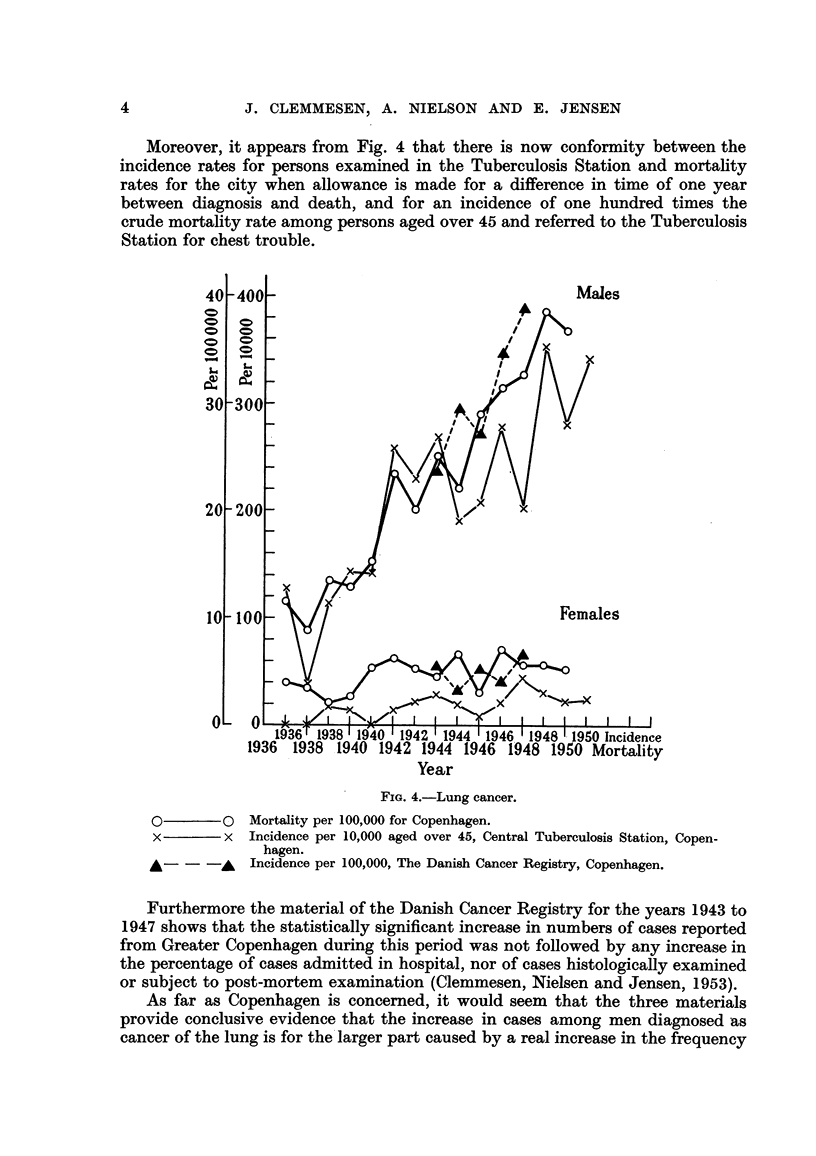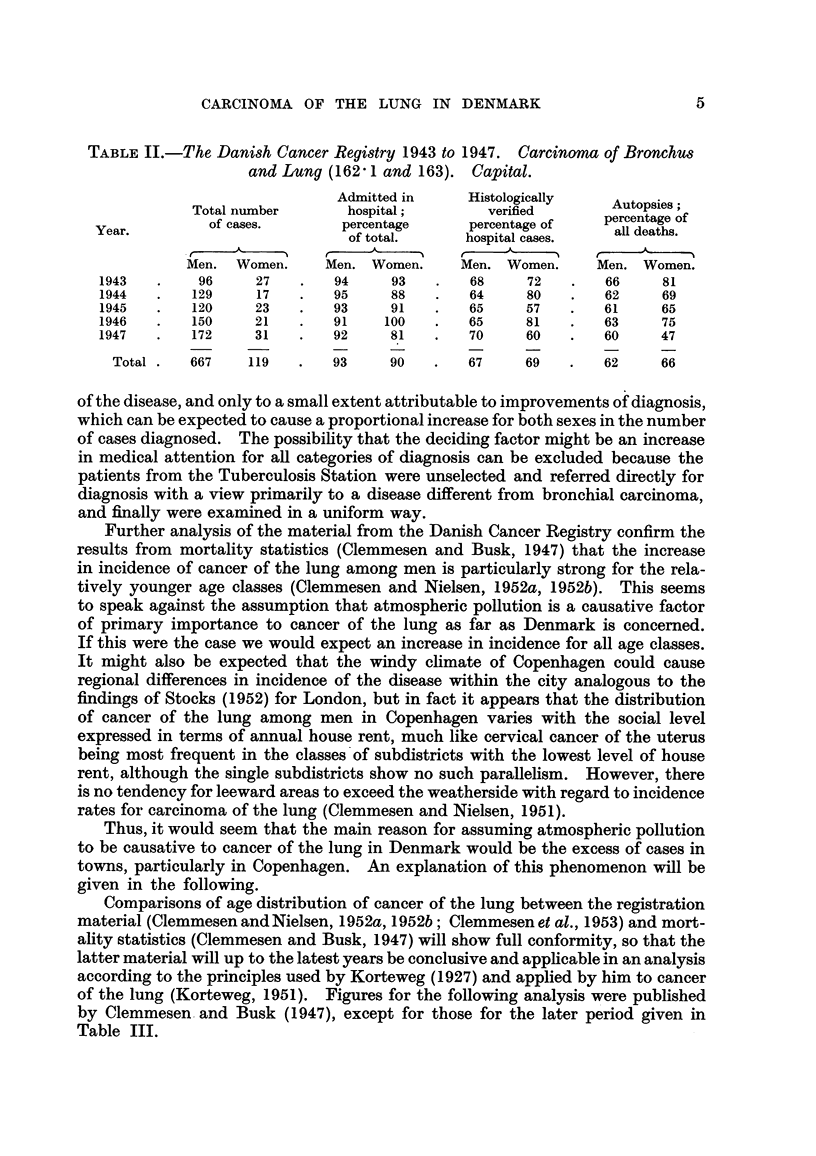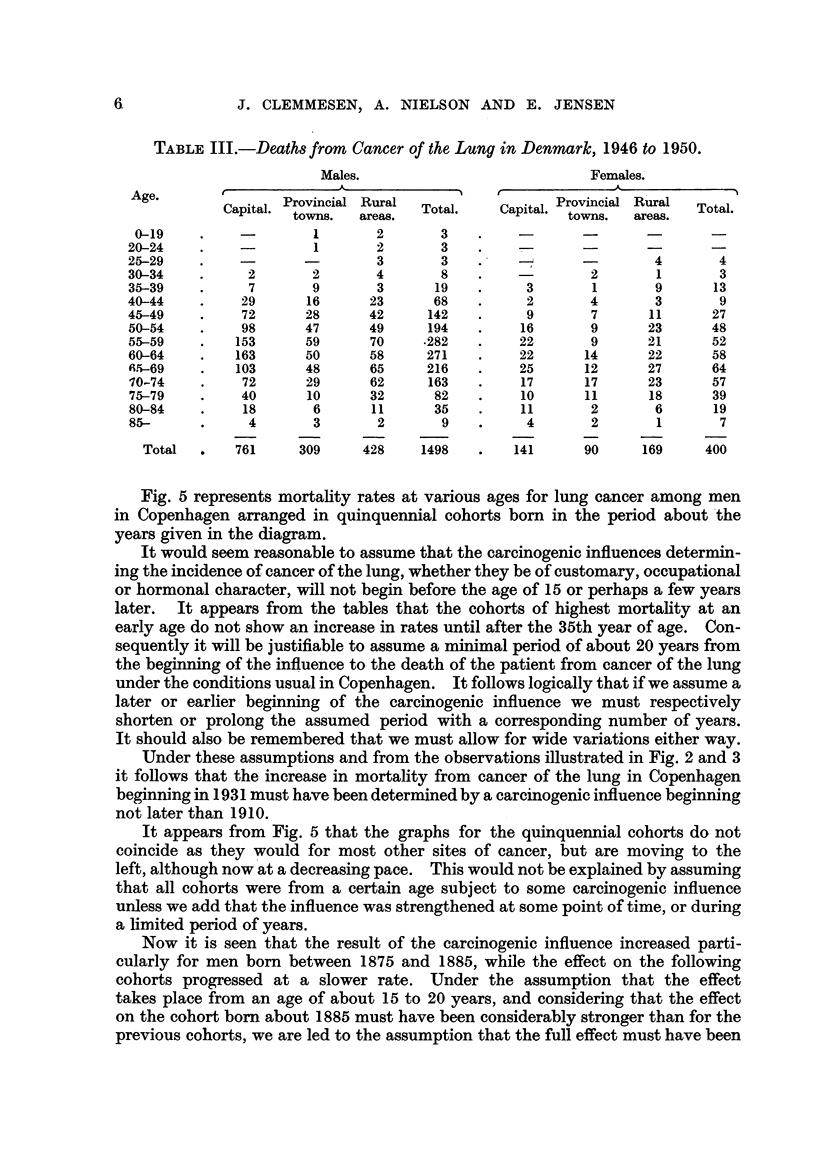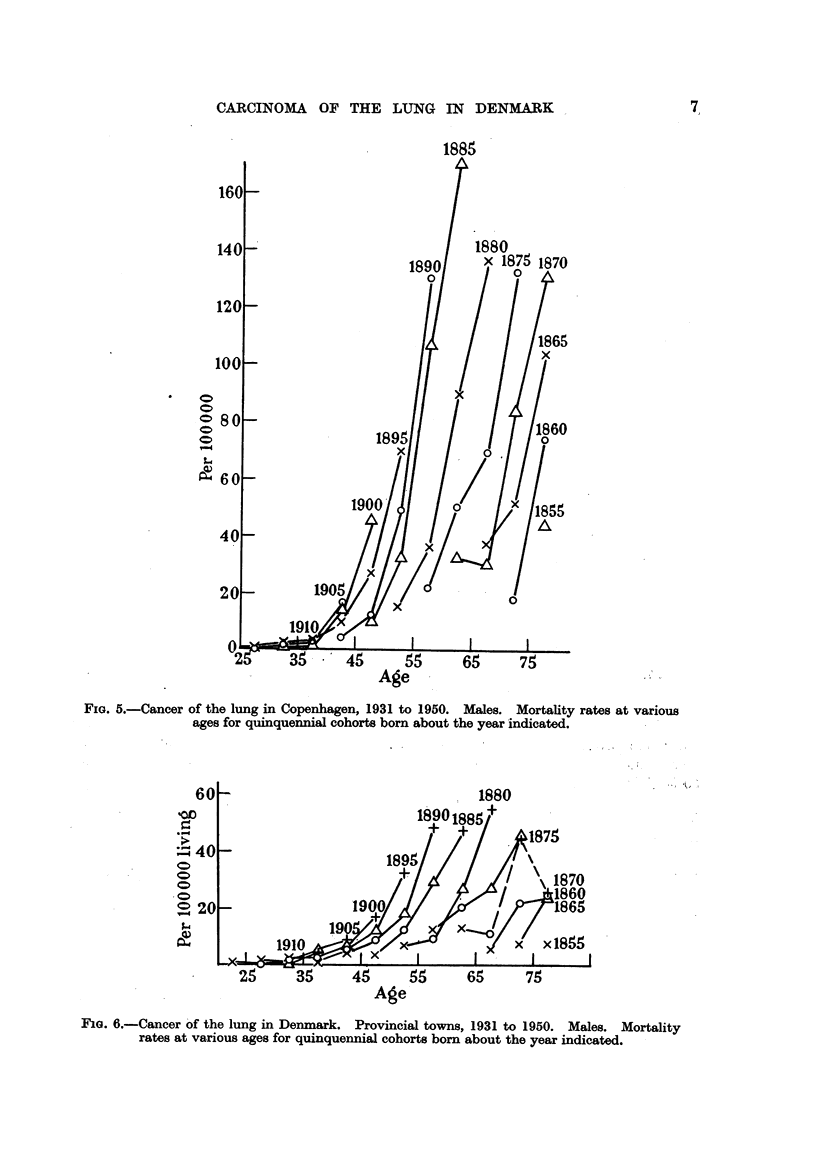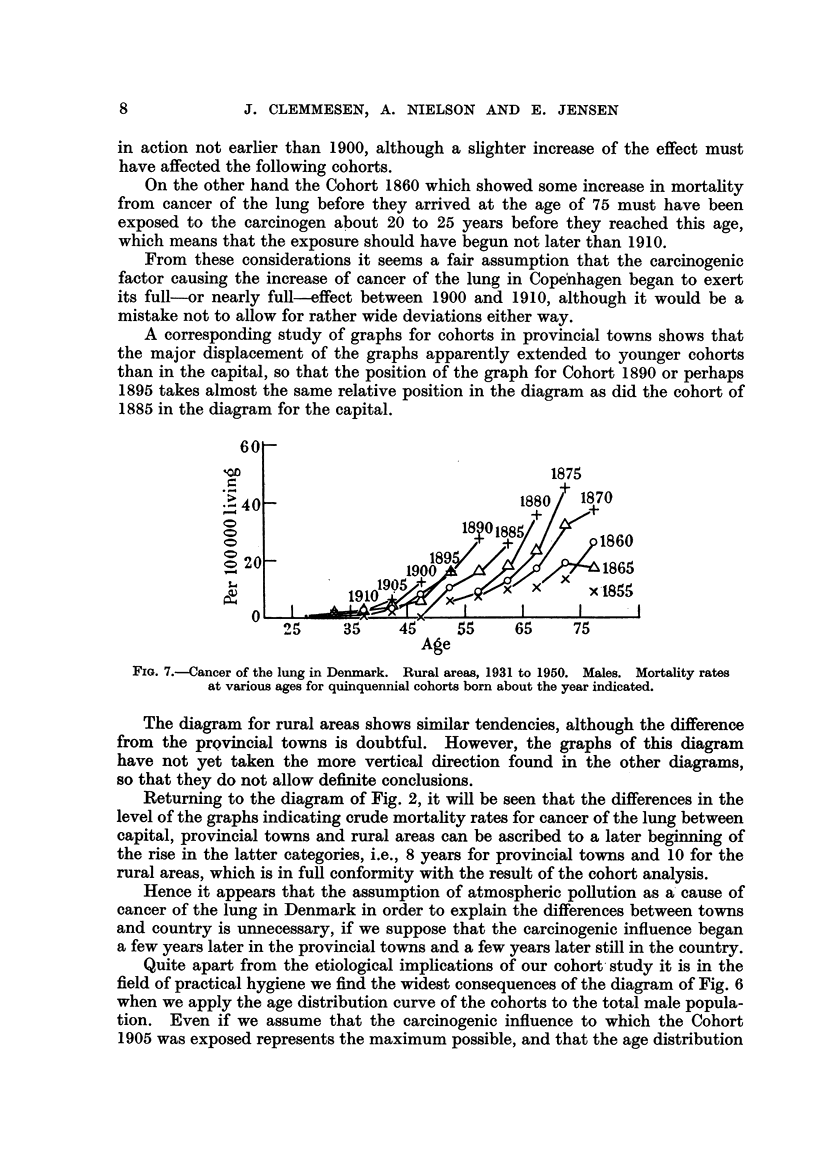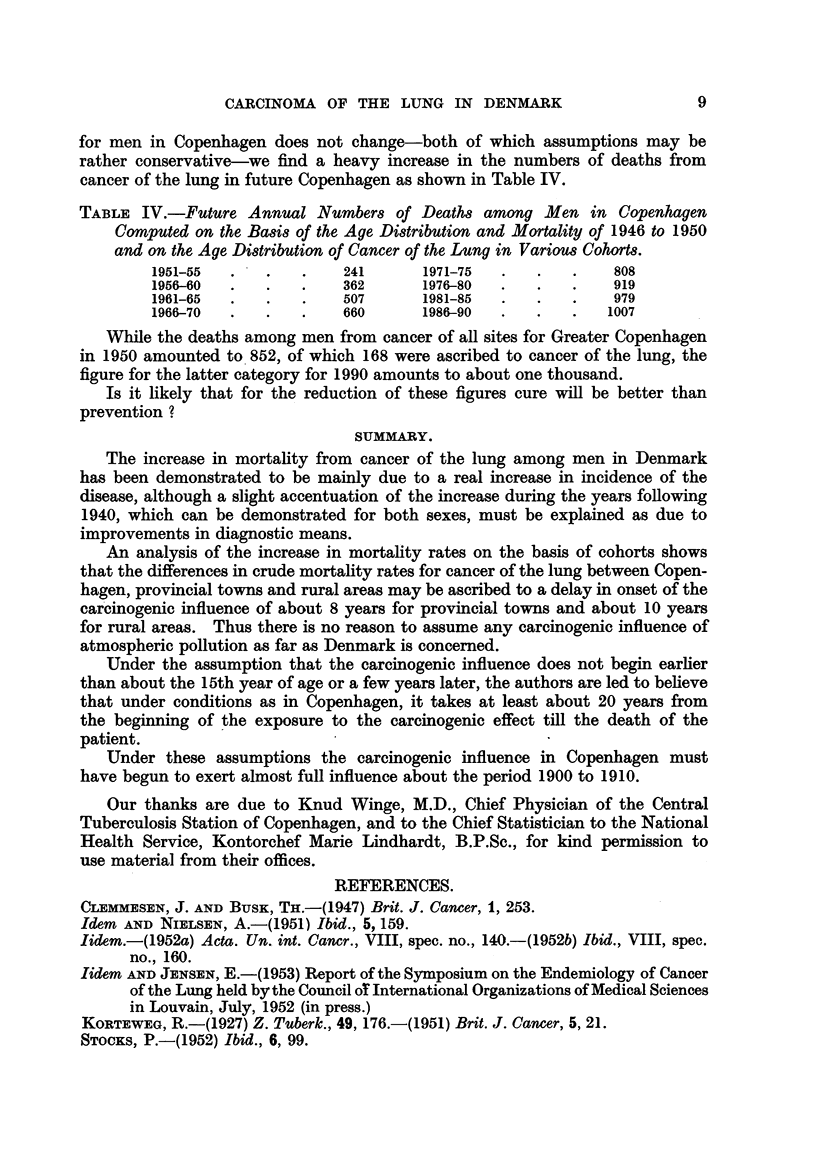# The Increase in Incidence of Carcinoma of the Lung in Denmark, 1931 to 1950

**DOI:** 10.1038/bjc.1953.1

**Published:** 1953-03

**Authors:** J. Clemmesen, A. Nielsen, E. Jensen


// BRITISH JOURNAL OF CANCER

VOL. VII MARCH, 1953 NO. 1

### THE INCREASE IN INCIDENCE OF CARCINOMA OF THE LUNG IN DENMARK, 1931 TO 1950.

J. CLEMMESEN, A. NIELSEN, AND E. JENSEN.

*From the Danish Cancer Registry under the National Anti-Cancer League, Köbenhavn Ö.*

Received for publication January 31, 1953.

IN 1947 Clemmesen and Busk reviewed the material of the Danish death certificates with a diagnosis of cancer of the lung from the years 1931 to 1945 (Clemmesen and Busk, 1947). They found an increase in the numbers for males, particularly pronounced in Copenhagen, the capital, less striking in provincial towns, and somewhat smaller for rural areas.

However, the incidence of cancer of the lung among persons subject to routine examination in the Central Tuberculosis Station for the City of Copenhagen did not reflect the heavy increase in mortality found in the city as a whole. An increase in the number of cases aged over 45 did occur, particularly in 1941, but as illustrated in Fig. 1 the values decreased to some extent during the following years. The authors therefore concluded that no increase of the incidence of lung cancer corresponding in gravity to the tripling of the mortality rates for males

TABLE I.—*Cancer of Lung. (Revised figures.) Central Tuberculosis Station, Copenhagen.*

| Year. | Males aged over 45. Cancer. | Males aged over 45. Morbidity rate per 10,000. | Females aged over 45. Cancer. | Females aged over 45. Morbidity rate per 10,000. |
|---|---|---|---|---|
| 1936 | 6 | 128·8 | 0 | 0·0 |
| 1937 | 2 | 38·2 | 0 | 0·0 |
| 1938 | 6 | 114·1 | 1 | 17·2 |
| 1939 | 9 | 142·9 | 1 | 15·0 |
| 1940 | 8 | 140·8 | 0 | 0·0 |
| 1941 | 18 | 258·6 | 1 | 14·5 |
| 1942 | 21 | 229·3 | 2 | 22·8 |
| 1943 | 26 | 268·9 | 3 | 28·4 |
| 1944 | 20 | 189·4 | 1 | 9·8 |
| 1945 | 22 | 208·1 | 1 | 8·9 |
| 1946 | 43 | 278·3 | 3 | 21·6 |
| 1947 | 34 | 202·5 | 7 | 44·4 |
| 1948 | 44 | 353·1 | 4 | 30·8 |
| 1949 | 37 | 280·7 | 3 | 22·8 |
| 1950 | 54 | 342·6 | 4 | 25·5 |

in the capital from 1936 to 1945 was found among the patients of the Central Tuberculosis Station. For this reason it seemed to Clemmesen and Busk (1947) that the apparent increase in lung cancer mortality was to a very large extent conditioned by improvements in diagnostic means. It should be noted that during the years following 1940 lung surgery and systematic bronchoscopy were introduced in Copenhagen, and that the mortality rates for cancer of the lung increased particularly steeply during this period following the increase of cases found in the Tuberculosis Station with a delay of one year.

However, seen in the light of later experience the picture has changed.

Fig. 1.—Lung cancer in Copenhagen, Central Tuberculosis Station. Incidence per 10,000 examined. Aged over 45.

O- - - - - - -O Material of 1947.

×———— × Revised material.

Tracing the ultimate fate of all its patients the Central Tuberculosis Station has been able retrospectively to diagnose a number of cases of cancer of the lung, so that the graph illustrating the incidence rates in its material should be changed, as shown in Fig. 1.

During the years up to 1950 the increase in mortality rates for cancer of the lung among men in the various areas of Denmark continued, and from Fig. 2 it appears that the increase following 1940, however steep, was not mainly due to the improvements in diagnostic technique taking place at this time. As we now can see from the slope of the later part of the graph these improvements caused just an acceleration of a continuous increase. Correspondingly, the smaller irregularity occurring in the graph for women (Fig. 3) at the same time now can be computed to be a statistically significant elevation of this, otherwise horizontal, graph, which is just what would be expected as result of an improvement in diagnostic technique.

FIG. 2.—Cancer of the lung in Denmark, 1931 to 1950. Mortality per 100,000 males.

X———X Greater Copenhagen.

O— — —O Provincial towns.

△- - - - - - -△ Rural areas.

FIG. 3.—Cancer of the lung in Denmark, 1931 to 1950. Mortality per 100,000 females.

X———X Greater Copenhagen.

O— — —O Provincial towns.

△- - - - - - -△ Rural areas.

Moreover, it appears from Fig. 4 that there is now conformity between the incidence rates for persons examined in the Tuberculosis Station and mortality rates for the city when allowance is made for a difference in time of one year between diagnosis and death, and for an incidence of one hundred times the crude mortality rate among persons aged over 45 and referred to the Tuberculosis Station for chest trouble.

Fig. 4.—Lung cancer.

○———○ Mortality per 100,000 for Copenhagen.
×———× Incidence per 10,000 aged over 45, Central Tuberculosis Station, Copenhagen.
▲— — —▲ Incidence per 100,000, The Danish Cancer Registry, Copenhagen.

Furthermore the material of the Danish Cancer Registry for the years 1943 to 1947 shows that the statistically significant increase in numbers of cases reported from Greater Copenhagen during this period was not followed by any increase in the percentage of cases admitted in hospital, nor of cases histologically examined or subject to post-mortem examination (Clemmesen, Nielsen and Jensen, 1953).

As far as Copenhagen is concerned, it would seem that the three materials provide conclusive evidence that the increase in cases among men diagnosed as cancer of the lung is for the larger part caused by a real increase in the frequency

TABLE II.—*The Danish Cancer Registry* 1943 *to* 1947. *Carcinoma of Bronchus and Lung* (162·1 *and* 163). *Capital.*

| Year. | Total number of cases. | | Admitted in hospital; percentage of total. | | Histologically verified percentage of hospital cases. | | Autopsies; percentage of all deaths. | |
|---|---|---|---|---|---|---|---|---|
| | Men. | Women. | Men. | Women. | Men. | Women. | Men. | Women. |
| 1943 | 96 | 27 | 94 | 93 | 68 | 72 | 66 | 81 |
| 1944 | 129 | 17 | 95 | 88 | 64 | 80 | 62 | 69 |
| 1945 | 120 | 23 | 93 | 91 | 65 | 57 | 61 | 65 |
| 1946 | 150 | 21 | 91 | 100 | 65 | 81 | 63 | 75 |
| 1947 | 172 | 31 | 92 | 81 | 70 | 60 | 60 | 47 |
| Total | 667 | 119 | 93 | 90 | 67 | 69 | 62 | 66 |

of the disease, and only to a small extent attributable to improvements of diagnosis, which can be expected to cause a proportional increase for both sexes in the number of cases diagnosed. The possibility that the deciding factor might be an increase in medical attention for all categories of diagnosis can be excluded because the patients from the Tuberculosis Station were unselected and referred directly for diagnosis with a view primarily to a disease different from bronchial carcinoma, and finally were examined in a uniform way.

Further analysis of the material from the Danish Cancer Registry confirm the results from mortality statistics (Clemmesen and Busk, 1947) that the increase in incidence of cancer of the lung among men is particularly strong for the relatively younger age classes (Clemmesen and Nielsen, 1952*a*, 1952*b*). This seems to speak against the assumption that atmospheric pollution is a causative factor of primary importance to cancer of the lung as far as Denmark is concerned. If this were the case we would expect an increase in incidence for all age classes. It might also be expected that the windy climate of Copenhagen could cause regional differences in incidence of the disease within the city analogous to the findings of Stocks (1952) for London, but in fact it appears that the distribution of cancer of the lung among men in Copenhagen varies with the social level expressed in terms of annual house rent, much like cervical cancer of the uterus being most frequent in the classes of subdistricts with the lowest level of house rent, although the single subdistricts show no such parallelism. However, there is no tendency for leeward areas to exceed the weatherside with regard to incidence rates for carcinoma of the lung (Clemmesen and Nielsen, 1951).

Thus, it would seem that the main reason for assuming atmospheric pollution to be causative to cancer of the lung in Denmark would be the excess of cases in towns, particularly in Copenhagen. An explanation of this phenomenon will be given in the following.

Comparisons of age distribution of cancer of the lung between the registration material (Clemmesen and Nielsen, 1952*a*, 1952*b*; Clemmesen *et al.*, 1953) and mortality statistics (Clemmesen and Busk, 1947) will show full conformity, so that the latter material will up to the latest years be conclusive and applicable in an analysis according to the principles used by Korteweg (1927) and applied by him to cancer of the lung (Korteweg, 1951). Figures for the following analysis were published by Clemmesen and Busk (1947), except for those for the later period given in Table III.

TABLE III.—*Deaths from Cancer of the Lung in Denmark,* 1946 *to* 1950.

| Age. | Males. | | | | Females. | | | |
|---|---|---|---|---|---|---|---|---|
| | Capital. | Provincial towns. | Rural areas. | Total. | Capital. | Provincial towns. | Rural areas. | Total. |
| 0–19 | — | 1 | 2 | 3 | — | — | — | — |
| 20–24 | — | 1 | 2 | 3 | — | — | — | — |
| 25–29 | — | — | 3 | 3 | — | — | 4 | 4 |
| 30–34 | 2 | 2 | 4 | 8 | — | 2 | 1 | 3 |
| 35–39 | 7 | 9 | 3 | 19 | 3 | 1 | 9 | 13 |
| 40–44 | 29 | 16 | 23 | 68 | 2 | 4 | 3 | 9 |
| 45–49 | 72 | 28 | 42 | 142 | 9 | 7 | 11 | 27 |
| 50–54 | 98 | 47 | 49 | 194 | 16 | 9 | 23 | 48 |
| 55–59 | 153 | 59 | 70 | ·282 | 22 | 9 | 21 | 52 |
| 60–64 | 163 | 50 | 58 | 271 | 22 | 14 | 22 | 58 |
| 65–69 | 103 | 48 | 65 | 216 | 25 | 12 | 27 | 64 |
| 70–74 | 72 | 29 | 62 | 163 | 17 | 17 | 23 | 57 |
| 75–79 | 40 | 10 | 32 | 82 | 10 | 11 | 18 | 39 |
| 80–84 | 18 | 6 | 11 | 35 | 11 | 2 | 6 | 19 |
| 85– | 4 | 3 | 2 | 9 | 4 | 2 | 1 | 7 |
| Total | 761 | 309 | 428 | 1498 | 141 | 90 | 169 | 400 |

Fig. 5 represents mortality rates at various ages for lung cancer among men in Copenhagen arranged in quinquennial cohorts born in the period about the years given in the diagram.

It would seem reasonable to assume that the carcinogenic influences determining the incidence of cancer of the lung, whether they be of customary, occupational or hormonal character, will not begin before the age of 15 or perhaps a few years later. It appears from the tables that the cohorts of highest mortality at an early age do not show an increase in rates until after the 35th year of age. Consequently it will be justifiable to assume a minimal period of about 20 years from the beginning of the influence to the death of the patient from cancer of the lung under the conditions usual in Copenhagen. It follows logically that if we assume a later or earlier beginning of the carcinogenic influence we must respectively shorten or prolong the assumed period with a corresponding number of years. It should also be remembered that we must allow for wide variations either way.

Under these assumptions and from the observations illustrated in Fig. 2 and 3 it follows that the increase in mortality from cancer of the lung in Copenhagen beginning in 1931 must have been determined by a carcinogenic influence beginning not later than 1910.

It appears from Fig. 5 that the graphs for the quinquennial cohorts do not coincide as they would for most other sites of cancer, but are moving to the left, although now at a decreasing pace. This would not be explained by assuming that all cohorts were from a certain age subject to some carcinogenic influence unless we add that the influence was strengthened at some point of time, or during a limited period of years.

Now it is seen that the result of the carcinogenic influence increased particularly for men born between 1875 and 1885, while the effect on the following cohorts progressed at a slower rate. Under the assumption that the effect takes place from an age of about 15 to 20 years, and considering that the effect on the cohort born about 1885 must have been considerably stronger than for the previous cohorts, we are led to the assumption that the full effect must have been

FIG. 5.—Cancer of the lung in Copenhagen, 1931 to 1950. Males. Mortality rates at various ages for quinquennial cohorts born about the year indicated.

FIG. 6.—Cancer of the lung in Denmark. Provincial towns, 1931 to 1950. Males. Mortality rates at various ages for quinquennial cohorts born about the year indicated.

in action not earlier than 1900, although a slighter increase of the effect must have affected the following cohorts.

On the other hand the Cohort 1860 which showed some increase in mortality from cancer of the lung before they arrived at the age of 75 must have been exposed to the carcinogen about 20 to 25 years before they reached this age, which means that the exposure should have begun not later than 1910.

From these considerations it seems a fair assumption that the carcinogenic factor causing the increase of cancer of the lung in Copenhagen began to exert its full—or nearly full—effect between 1900 and 1910, although it would be a mistake not to allow for rather wide deviations either way.

A corresponding study of graphs for cohorts in provincial towns shows that the major displacement of the graphs apparently extended to younger cohorts than in the capital, so that the position of the graph for Cohort 1890 or perhaps 1895 takes almost the same relative position in the diagram as did the cohort of 1885 in the diagram for the capital.

FIG. 7.—Cancer of the lung in Denmark. Rural areas, 1931 to 1950. Males. Mortality rates at various ages for quinquennial cohorts born about the year indicated.

The diagram for rural areas shows similar tendencies, although the difference from the provincial towns is doubtful. However, the graphs of this diagram have not yet taken the more vertical direction found in the other diagrams, so that they do not allow definite conclusions.

Returning to the diagram of Fig. 2, it will be seen that the differences in the level of the graphs indicating crude mortality rates for cancer of the lung between capital, provincial towns and rural areas can be ascribed to a later beginning of the rise in the latter categories, i.e., 8 years for provincial towns and 10 for the rural areas, which is in full conformity with the result of the cohort analysis.

Hence it appears that the assumption of atmospheric pollution as a cause of cancer of the lung in Denmark in order to explain the differences between towns and country is unnecessary, if we suppose that the carcinogenic influence began a few years later in the provincial towns and a few years later still in the country.

Quite apart from the etiological implications of our cohort study it is in the field of practical hygiene we find the widest consequences of the diagram of Fig. 6 when we apply the age distribution curve of the cohorts to the total male population. Even if we assume that the carcinogenic influence to which the Cohort 1905 was exposed represents the maximum possible, and that the age distribution for men in Copenhagen does not change—both of which assumptions may be rather conservative—we find a heavy increase in the numbers of deaths from cancer of the lung in future Copenhagen as shown in Table IV.

TABLE IV.—*Future Annual Numbers of Deaths among Men in Copenhagen Computed on the Basis of the Age Distribution and Mortality of* 1946 *to* 1950 *and on the Age Distribution of Cancer of the Lung in Various Cohorts.*

| | | | |
|---|---|---|---|
| 1951–55 . . . | 241 | 1971–75 . . . | 808 |
| 1956–60 . . . | 362 | 1976–80 . . . | 919 |
| 1961–65 . . . | 507 | 1981–85 . . . | 979 |
| 1966–70 . . . | 660 | 1986–90 . . . | 1007 |

While the deaths among men from cancer of all sites for Greater Copenhagen in 1950 amounted to 852, of which 168 were ascribed to cancer of the lung, the figure for the latter category for 1990 amounts to about one thousand.

Is it likely that for the reduction of these figures cure will be better than prevention ?

SUMMARY.

The increase in mortality from cancer of the lung among men in Denmark has been demonstrated to be mainly due to a real increase in incidence of the disease, although a slight accentuation of the increase during the years following 1940, which can be demonstrated for both sexes, must be explained as due to improvements in diagnostic means.

An analysis of the increase in mortality rates on the basis of cohorts shows that the differences in crude mortality rates for cancer of the lung between Copenhagen, provincial towns and rural areas may be ascribed to a delay in onset of the carcinogenic influence of about 8 years for provincial towns and about 10 years for rural areas. Thus there is no reason to assume any carcinogenic influence of atmospheric pollution as far as Denmark is concerned.

Under the assumption that the carcinogenic influence does not begin earlier than about the 15th year of age or a few years later, the authors are led to believe that under conditions as in Copenhagen, it takes at least about 20 years from the beginning of the exposure to the carcinogenic effect till the death of the patient.

Under these assumptions the carcinogenic influence in Copenhagen must have begun to exert almost full influence about the period 1900 to 1910.

Our thanks are due to Knud Winge, M.D., Chief Physician of the Central Tuberculosis Station of Copenhagen, and to the Chief Statistician to the National Health Service, Kontorchef Marie Lindhardt, B.P.Sc., for kind permission to use material from their offices.

### REFERENCES.

CLEMMESEN, J. AND BUSK, TH.—(1947) *Brit. J. Cancer*, **1**, 253.

*Idem* AND NIELSEN, A.—(1951) *Ibid.*, **5**, 159.

*Iidem.*—(1952*a*) *Acta. Un. int. Cancr.*, VIII, spec. no., 140.—(1952*b*) *Ibid.*, VIII, spec. no., 160.

*Iidem* AND JENSEN, E.—(1953) Report of the Symposium on the Endemiology of Cancer of the Lung held by the Council of International Organizations of Medical Sciences in Louvain, July, 1952 (in press.)

KORTEWEG, R.—(1927) *Z. Tuberk.*, **49**, 176.—(1951) *Brit. J. Cancer*, **5**, 21.

STOCKS, P.—(1952) *Ibid.*, **6**, 99.